# *Clostridium perfringens* Bacteraemia following Normal Vaginal Delivery

**DOI:** 10.1155/2024/1545571

**Published:** 2024-07-09

**Authors:** Mokhamad Zhaffal, Anastasia Salame, Johnny Awwad

**Affiliations:** ^1^ Obstetrics and Gynecology Department American University of Beirut, Beirut, Lebanon; ^2^ Reproductive Endocrinology and Infertility Unit Obstetrics and Gynecology Department American University of Beirut, Beirut, Lebanon

## Abstract

Clostridial uterine infections and bacteraemia are of a rare occurrence, especially in the absence of risk factors. However, when encountered, they can carry significant morbidity and mortality rates. We present a rare case of *C. perfringens* bacteraemia in the immediate postpartum period of a noncomplicated vaginal delivery. Prompt diagnosis and early initiation of treatment were imperative for ensuring a safe recovery of the patient. Despite the fact that bacteraemia caused by *C. perfringens* is an infrequent event in the uneventful postpartum phase, maintaining vigilance for the potential occurrence of such an event allows for early detection and timely administration of antibiotics and resuscitative measures.

## 1. Introduction

*Clostridium perfringens*, an anaerobic, nonmotile, Gram-positive, spore-forming bacillus, is an integral component of the gastrointestinal and genitourinary flora, known for its pathogenicity. This bacillus is notorious for releasing various exotoxins with remarkable enteroinvasive properties [1]. The clinical spectrum associated with *Clostridium perfringens* infections is broad, ranging from cellulitis, necrotising fasciitis, and myonecrosis to acute hemolysis [2].

Bacteraemia stemming from *C. perfringens* is relatively infrequent in hospital settings, with reported incidences ranging from 0.04 to 2% [1, 3]. The clinical manifestations often lack specificity, resulting in delayed diagnoses and treatment initiation. Cases of bacteraemia are primarily linked to compromised immunity, such as in cancer and immunosuppressed patients [3]. A noteworthy association has been proposed between *C. perfringens* bacteraemia and gastrointestinal tract malignancies [3, 4]. Despite the source of bacteraemia, documented cases exhibit an elevated mortality rate, reaching 15.2% [1].

Pregnancy-related *C. perfringens* infections are associated with uterine instrumentation, caesarean sections, and abortions. The presence of a leiomyoma in the context of an abortion appears to heighten the risk of soft tissue clostridial infections [5]. Regardless of predisposing factors, Clostridial uterine infections and bacteraemia carry significant morbidity and mortality rates. Therefore, prompt diagnosis and early initiation of treatment are imperative for ensuring a safe recovery [5].

In the realm of our discourse, we bring forth a distinctive case, elucidating an occurrence of *Clostridium perfringens* bacteraemia within the immediate postpartum period following a vaginal delivery, characterised by the absence of discernible underlying risk factors or predisposing medical conditions. This singular case serves as a poignant reminder of the intricate and often unpredictable nature of *C. perfringens* infections, urging a comprehensive understanding of the diverse clinical manifestations and potential risk factors associated with this pathogen. The scarcity of instances featuring *C. perfringens* bacteraemia in the absence of identifiable risk factors underscores the need for meticulous investigation into the nuanced dynamics of this infection.

In conclusion, the singular case presented here serves as a microcosm of the broader challenges and opportunities in understanding and managing *C. perfringens* infections. As the landscape of medical research continues to evolve, the synergy between clinical observations and scientific inquiry remains paramount in guiding clinicians towards more effective strategies for the diagnosis, management, and prevention of *Clostridium perfringens* infections.

## 2. Case Description

A 40-year-old woman presented with chills, fever, and abdominal pain forty hours following an uncomplicated vaginal delivery. Her antenatal course transpired without any noteworthy events, culminating in a vaginal delivery at 38 + 3 weeks of gestation. The first stage of labour unfolded smoothly over 13 hours, marked by the artificial rupture of membranes at the 11th hour, resulting in clear amniotic fluid. The subsequent second stage, lasting 2 hours, encountered a solitary episode of low-grade fever (38 degrees Celsius), which spontaneously resolved. The noninstrumental vaginal delivery yielded a live-born male singleton weighing 3,245 grams, with Apgar scores of 9 and 9 at 1 and 5 minutes, respectively. Following a midline second-degree laceration, primarily repaired, the placenta was spontaneously and completely delivered.

The patient's medical history included a hepatitis B carrier state with consistently negative viral loads during pregnancy and a past surgical history featuring inguinal hernia repair. Notably, she had experienced first-trimester recurrent pregnancy loss and recurrent implantation failure, achieving pregnancy after thirteen unsuccessful in vitro fertilization (IVF) attempts.

Upon presentation, vital signs revealed a temperature of 38.90°C and tachycardia peaking at 101 bpm, with overall hemodynamic stability. Physical examination was unremarkable except for significant tenderness at the perineal repair site. Relevant laboratory testing indicated a white blood cell count of 16,700 cells/mm^3^ (83% polymorphonuclear cells) and positive urine leucocyte esterase, prompting the initiation of ceftriaxone for a presumed urinary tract infection.

Eight hours postcollection, blood cultures revealed the presence of *C. perfringens*. Consequently, the antibiotic regimen was adjusted to Tazocin (piperacillin/tazobactam) 4.5 grams intravenously every 6 hours and metronidazole 500 mg every 8 hours. Abdominopelvic computed tomography showed no evidence of intraabdominal tumours, perineal fasciitis, or collections. Following five days of antibiotic treatment, her symptoms resolved completely, leading to her discharge home on amoxicillin/clavulanic acid and metronidazole. A week after discharge, during a clinic follow-up, all signs and symptoms had resolved completely, reflecting the successful resolution of the *C. perfringens* infection. This case underscores the necessity for detailed exploration of clinical scenarios, especially in the context of atypical presentations, contributing to a broader understanding of the complexities surrounding *Clostridium perfringens* infections and guiding effective clinical management strategies.

## 3. Discussion

*C. perfringens,* an anaerobic bacterium previously known as *Clostridium welchii*, has been sporadically isolated from vaginal and cervical fluids in seemingly healthy and asymptomatic women [6]. While generally benign, this anaerobic bacterium can, in rare instances, precipitate postpartum bacteraemia, presenting a diagnostic challenge due to its infrequency. Smith et al. reported recovering *C. perfringens* from 10 to 27% of genital tract cultures in aborting women, with sepsis manifesting in less than 1% of the cases [7]. The exploration of *C. perfringens* has seen notable advancements in the recent past, contributing valuable insights into its epidemiology, pathogenic mechanisms, and diagnostic approaches.

In our presented case, the patient had a midline second-degree laceration. Second-degree lacerations are limited to the vaginal wall and do not reach the rectal cavity, hence less likely to predispose to bacterial crossover from the rectal space to vaginal and or uterine space. The absence of identifiable risk factors for *C. perfringens* bacteraemia led to a positive response to broad-spectrum antibiotics and appropriate resuscitative measures. Clinical manifestations arise from the release of twelve exotoxins, with alpha toxin, responsible for platelet aggregation, reduced blood flow, and soft tissue necrosis, identified as the most crucial. Timely administration of antibiotic therapy, resuscitative measures, and surgical intervention, involving the resection of affected tissues, are critical in preventing life-threatening complications [8].

Lopez-Fabal et al. place emphasis on its more frequent occurrence in patients with compromised immunity, often associated with malignancies, diabetes mellitus, and advanced age [3]. In some instances, *Clostridium* bacteraemia may serve as the revealing event of underlying diseases, as reported by a Spanish group in a fatal case where postmortem analysis unveiled an undiagnosed colorectal neoplasm [9]. Coincidental diagnoses of gynaecologic malignancies, including undifferentiated uterine sarcoma and uterine endometrial adenocarcinoma, have also been reported [10]. Importantly, our patient showed no evidence of any gastrointestinal disorders. The patient did have a history of recurrent pregnancy losses as well as recurrent implantation failure. It is not clear whether *C. perfringens* colonisation predisposes to adverse reproductive and antenatal outcomes as to the best of our knowledge, there are no published data on the topic. There is a rising evidence highlighting the effect of the endometrial microbiome on the fertility potential and reproductive outcomes, yet *C. perfringens* was not found as one of the culprits [11].

The literature on postpartum *C. perfringens*-induced bacteraemia cases in previously healthy patients is somewhat limited, making risk factors in obstetrical patients elusive. Possible contributors could include the presence of leiomyoma, uterine instrumentation, caesarean section, abortion, prolonged labour, and traumatic delivery [5]. Typically, treatment involves broad-spectrum antibiotics with intravenous hydration, and surgical debridement is considered if no clinical improvement is documented.

In summary, *C. perfringens* bacteraemia during the postpartum period, though rare, demands vigilant consideration. As exemplified in our case report, diagnosis and management are paramount, with blood cultures forming a crucial diagnostic tool for patients presenting with postdelivery fever and sepsis. The intricate interplay between this pathogen and obstetrical risk factors necessitates ongoing research and clinical vigilance. Understanding the diverse clinical scenarios associated with *C. perfringens* infections is essential for optimising therapeutic strategies and preventive measures.

## 4. Conclusion

Bacteraemia caused by *C. perfringens* is an infrequent event in the uneventful postpartum phase. Maintaining vigilance for the potential occurrence of such an event allows for early detection and timely administration of antibiotics and resuscitative measures. Patients exhibiting postdelivery fever and sepsis in addition to blood culture analysis should undergo computerised tomography, which proves to be a suitable method for screening necrotising and gangrenous processes, along with detecting potential abdominopelvic malignancies.

## Data Availability

The datasets used and/or analysed during the current study are available from the corresponding author on reasonable request.
